# High permittivity, breakdown strength, and energy storage density of polythiophene-encapsulated BaTiO_3_ nanoparticles

**DOI:** 10.3762/bjnano.11.103

**Published:** 2020-08-10

**Authors:** Adnanullah Khan, Amir Habib, Adeel Afzal

**Affiliations:** 1 School of Chemical and Materials Engineering, National University of Science and Technology, Sect. H-12, Islamabad, 44000, Pakistan; 2 Department of Physics, College of Science, University of Hafr Al Batin, PO Box 1803, Hafr Al Batin, 39524, Saudi Arabia; 3 Department of Chemistry, College of Science, University of Hafr Al Batin, PO Box 1803, Hafr Al Batin, 39524, Saudi Arabia

**Keywords:** barium titanate (BaTiO_3_) nanoparticles, breakdown strength, dielectric materials, energy storage, polythiophene

## Abstract

High permittivity and breakdown strength are desired to improve the energy storage density of dielectric materials based on reinforced polymer composites. This article presents the synthesis of polythiophene-encapsulated BaTiO_3_ (BTO-PTh) nanoparticles via an in situ Cu(II)-catalyzed chemical oxidative polymerization of thiophene monomer on hydrothermally obtained tetragonal BTO nanocrystals. The formed core–shell-type BTO-PTh nanoparticles exhibit excellent dielectric properties with high permittivity (25.2) and low loss (0.04) at high frequency (10^6^ Hz). A thick PTh encapsulation layer on the surface of the BTO nanoparticles improves their breakdown strength from 47 to 144 kV/mm and the energy storage density from 0.32 to 2.48 J/cm^3^. A 7.75-fold increase in the energy storage density of the BTO-PTh nanoparticles is attributed to simultaneously high permittivity and breakdown strength, which are excellent for potential energy storage applications.

## Introduction

The fast-paced progress and constantly growing demand of microelectronic devices and energy storage technologies have led to extensive research on the development of new dielectric materials [1–3]. High-κ ceramic-based dielectric materials such as BaTiO_3_ (BTO) have been prepared and used in actuators, capacitors, and communication devices [4–6]. However, the ceramic-based dielectrics are often brittle, and possess low electrical breakdown strength and energy storage density [7], which hampers their practical applications in energy storage devices such as dielectric capacitors. To overcome these problems, BTO-polymer composite-based dielectric materials have been developed and extensively investigated. Organic polymers offer many advantages including higher breakdown strength, lighter mass, greater flexibility, processability, and cost efficiency [8]. However, they are inherently poor dielectrics with very low permittivity (values in the range of 2–10) [2]. Thus, organic polymers are often reinforced with ceramic-based dielectric materials such as BTO.

The dielectric properties of BTO–polymer composites are known to improve considerably with the inclusion of BTO nanoparticles. For instance, Dang et al. [9] achieved a permittivity value of ca. 40 at 10 kHz with 50 vol % loading of BTO in polyvinylidene fluoride (PVDF). However, such a high content of BTO nanoparticles has severe effects on the overall performance of the composite dielectric materials [9–10], which are largely attributed to the interphase inhomogeneity, poor distribution, and agglomeration of BTO nanoparticles in the polymer matrix. You et al. [10] observed an increase in the permittivity (from 4 to 14) of poly(arylene ether nitrile) filled with 40 wt % polyaniline-functional-BTO nanoparticles, but they noticed that breakdown strength of the composite was critically affected at a high concentration of filler due to free charge accumulation at the interface.

Therefore, to improve breakdown strength and energy storage density of BTO, we propose the design of polythiophene (PTh)-encapsulated BaTiO_3_ nanoparticles with a 9:1 mass ratio of BTO/PTh, and a facile method for the synthesis of inverted [11] core–shell-type BTO-PTh nanostructures, which yields a uniform PTh coating on the BTO surface. BTO-PTh nanoparticles are prepared by Cu(II)-catalyzed oxidative polymerization of PTh on the BTO surface. This procedure yields a high BTO content in the PTh shell, which not only results in superior dielectric properties such as high permittivity and low loss, but also significantly increases breakdown strength. Consequently, core–shell BTO-PTh nanoparticles exhibit greatly improved energy storage density. We believe BTO-PTh nanoparticles are a promising material and this design is a noteworthy strategy for future research and advanced microelectronic and energy storage applications.

## Experimental

Barium hydroxide octahydrate (Ba(OH)_2_·8H_2_O, ≥98%, Sigma-Aldrich) and fine-grained titanium dioxide (TiO_2_ Tronox, 99.5%, Tronox Pigments GmbH) are used as Ba and Ti precursors for the hydrothermal synthesis of BTO nanoparticles. Ba(OH)_2_·8H_2_O also acts as the mineralizer and prevents the use of NaOH or KOH for controlling the pH value of the reaction mixture [12]. Equimolar amounts of Ba and Ti precursors are added to the double-distilled water in a PTFE-lined pressure vessel. The hydrothermal reaction is carried out in an autoclave (Berghof Instruments GmbH) at 120 °C and autogenous pressure for 24 h under stirring. The product is washed with dilute formic acid solution and double-distilled water to dissolve and remove impurities and is dried in an oven at 120 °C. The obtained dried product is pulverized to get BTO nanoparticles.

**Figure 1:**
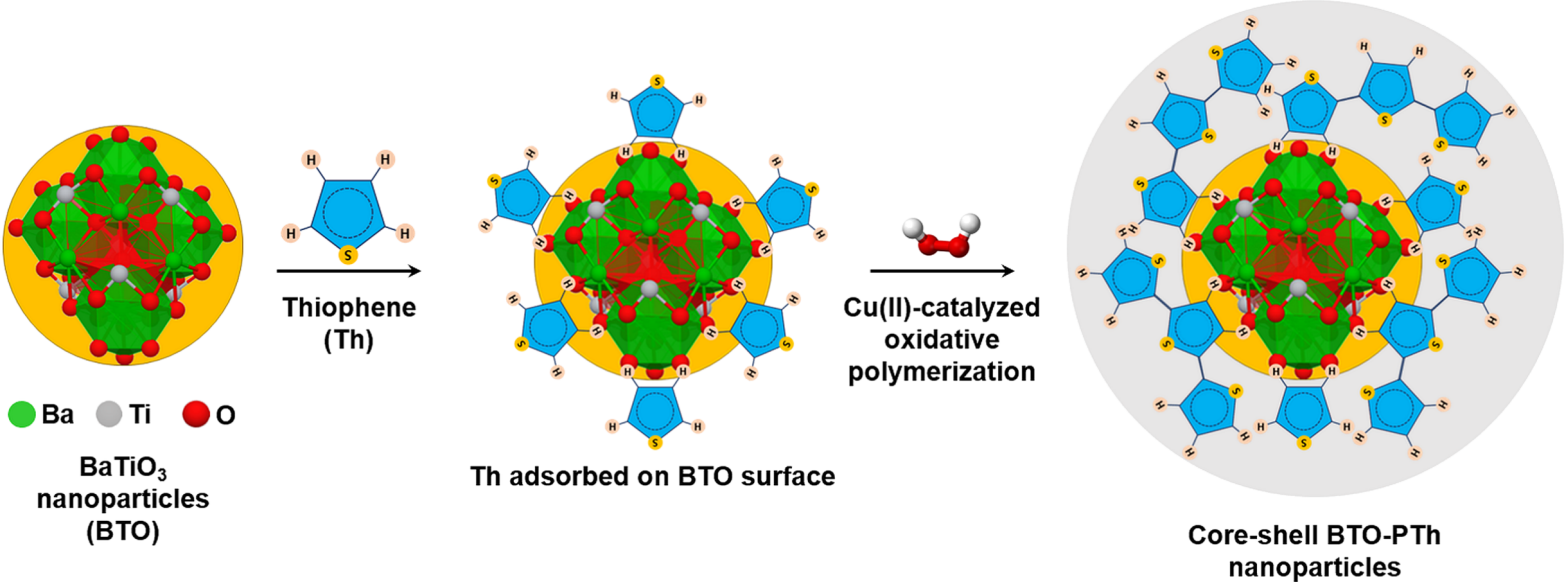
Schematic of the formation of core–shell BaTiO_3_-thiophene (BTO-PTh) nanoparticles. BTO nanoparticles and thiophene (Th) monomer are dispersed in water. Th-monomer molecules adsorb on the surface of BTO nanoparticles through weak intermolecular interactions. The chemical oxidative polymerization of Th initiated by H_2_O_2_ and catalyzed by Cu^2+^ ions yields the core–shell BTO-PTh nanoparticles.

In the next step, core–shell BTO-PTh nanoparticles are synthesized via in situ chemical oxidative polymerization of thiophene on hydrothermally produced BTO nanoparticles. BTO nanoparticles are first dispersed in double-distilled water containing sodium dodecyl sulfate (SDS, ACS reagent, ≥99.0%, Sigma Aldrich) and thiophene (Th, synthesis grade 99%, Scharlab, S.L.) monomer through ultrasonication for 1 h. The mass ratio of BTO/Th is adjusted to 9:1. Subsequently, hydrogen peroxide (H_2_O_2_, solution 30% w/w, Scharlab, S.L.) and copper(II) sulfate pentahydrate (CuSO_4_·5H_2_O, reagent grade, ≥98.0%, Honeywell) solution are added to the BTO-Th mixture. The reaction is performed by stirring the mixture for 7 h at 50 °C. The product is washed with double-distilled water and ethanol and is dried in a vacuum oven at 50 °C for 24 h. Figure 1 shows a schematic of the synthesis of core–shell BTO-PTh nanoparticles.

To compare dielectric properties and energy efficiency, a similar approach is used to prepare pristine polythiophene (PTh) without introducing BTO nanoparticles in the mixture. All materials including BTO, BTO-PTh, and PTh are characterized using Fourier-transform infrared spectroscopy (Nicolet 520 FTIR spectrophotometer) and X-ray diffraction (STOE STADI P X-ray diffractometer). The morphology of BTO and BTO-PTh nanoparticles is studied using scanning electron microscopy (JEOL JSM 6490LA SEM) and atomic force microscopy (JSPM-5200 scanning probe microscope). Electrical properties of the bulk materials are measured under ambient conditions with a Wayne Kerr 6505B precision impedance analyzer and a Hipotronics HD103 3kV DC Hipot Tester.

## Results and Discussion

**Figure 2:**
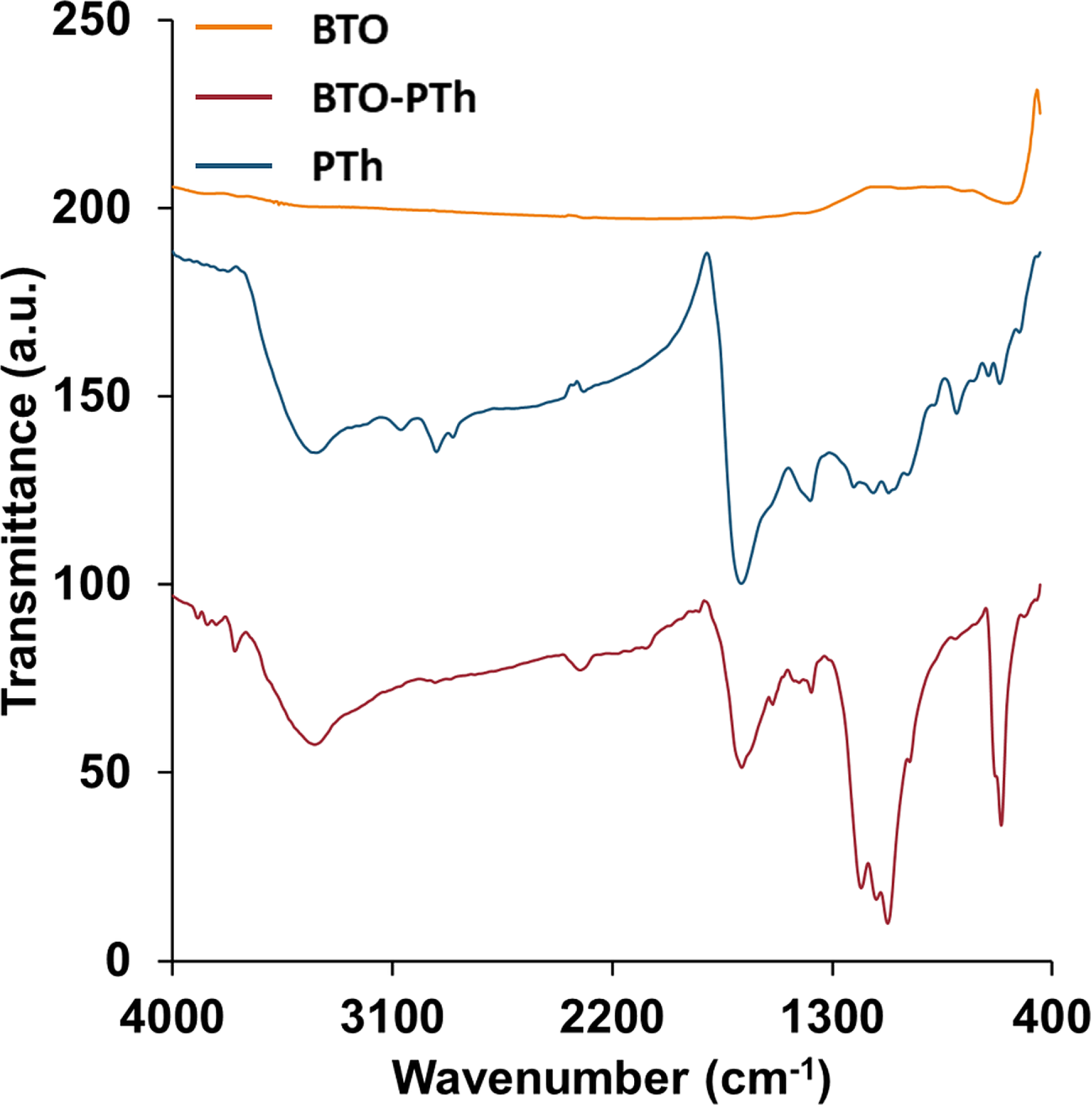
FTIR spectra of the as-prepared BTO nanoparticles, pristine PTh, and core–shell BTO-PTh nanoparticles.

Chemical structure and surface morphology of the as-prepared BTO nanoparticles, core–shell-type BTO-PTh nanoparticles, and pristine PTh are characterized and reported herein. Figure 2 shows the FTIR spectra of PTh, BTO, and BTO-PTh nanoparticles. A distinct peak at 560 cm^−1^ is the characteristic stretching vibration (νTi–O) of BTO structure [13–14]. PTh is characterized by the symmetric and asymmetric stretching vibrations (νC=C) of the aromatic ring at 1628 and 1385 cm^−1^, a sharp aromatic ring deformation (δC–S–C) at 610 cm^−1^, and a typical stretching vibration of the aromatic β-hydrogens (νC_β_–H) at 3060 cm^−1^ [15–16]. The broad transmittance peak at 3418 cm^−1^ and weak bands in the range of 2845–2935 cm^−1^ are attributed to the adsorbed water molecules (νO–H) and the carbonaceous impurities (νCH_3_, νCH_2_) in PTh, respectively. A peak at 1123 cm^−1^ that is also characteristic of PTh corresponds to the C_α_–C_α_ resonance absorption between two thiophene rings [17]. It demonstrates that PTh is predominantly formed by C_α_–C_α_ conjunction during the low-temperature oxidative polymerization. The transmittance peaks at 1035 cm^−1^ and 788 cm^−1^ further prove this point as they indicate the out-of-plane bending (τC_β_–H) and in-plane bending vibrations (ρC_β_–H) of PTh, respectively [17–18]. According to Wu et al. [17], the intensity of C_β_–H transmissions would considerably decrease if PTh had C_β_–C_β_ conjunction. In BTO-PTh spectrum, the out-of-plane and in-plane bending of C_β_–H shift to 1077 cm^−1^ and 983 cm^−1^, respectively, which is attributed to the interactions between β-hydrogens of PTh and oxygen atoms on the BTO surface.

**Figure 3:**
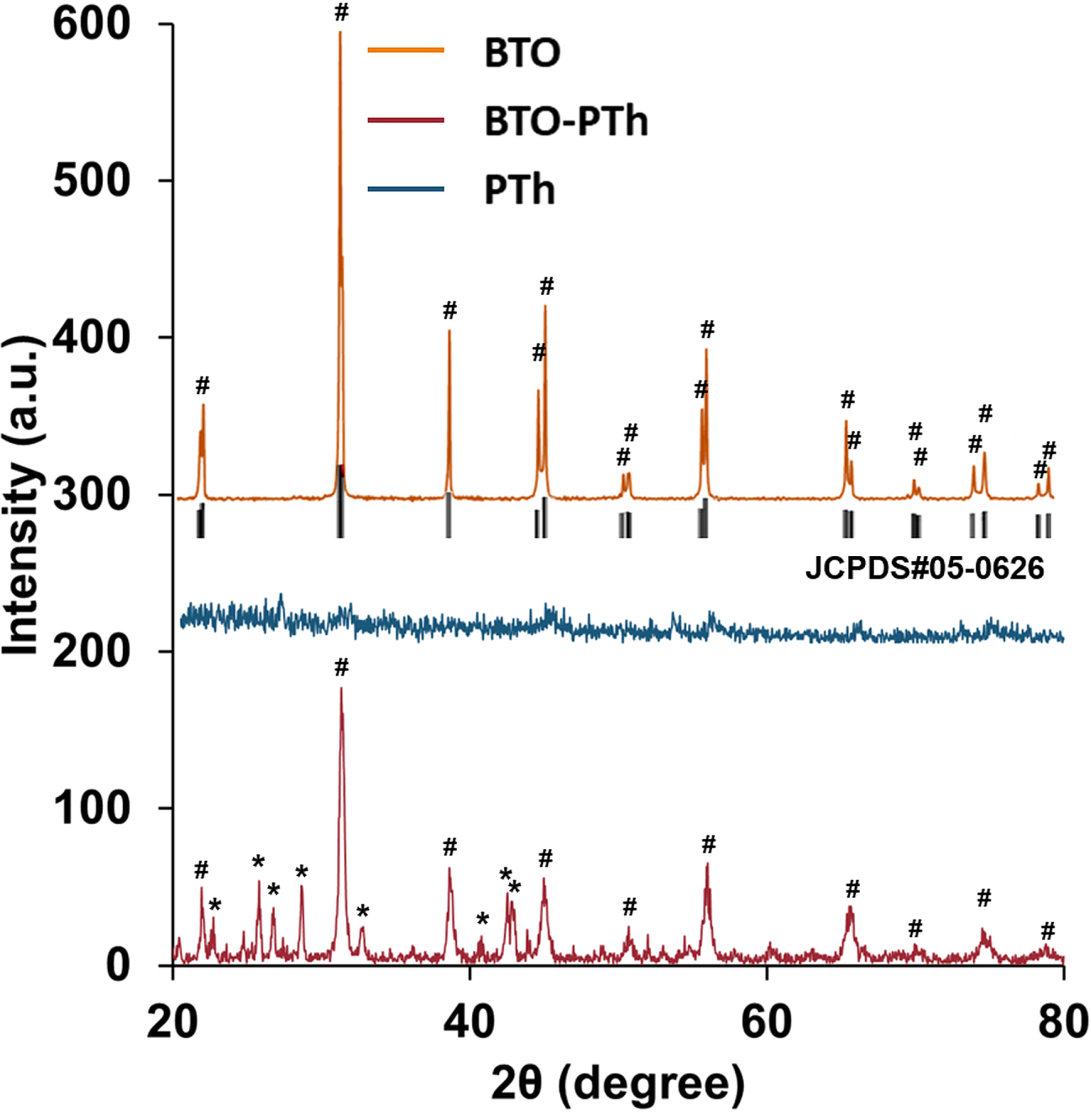
XRD patterns of the as-prepared BTO nanoparticles, pristine PTh, and core–shell BTO-PTh nanoparticles. The diffraction peaks denoted by (#) correspond to the tetragonal BTO lattice (JCPDS No. 05-0626), while peaks denoted by (*) correspond to the orthorhombic BaSO_4_ impurities.

X-ray diffraction patterns of BTO, PTh, and core–shell BTO-PTh nanoparticles are presented in Figure 3. Pristine PTh is amorphous in nature and shows a low-intensity broad peak at around 23°, which corresponds to the intermolecular π–π stacking structure and amorphous packing of the polymer [19]. The XRD pattern of hydrothermally prepared BTO nanoparticles shows good agreement with the tetragonal perovskite structure (JCPDS No. 05-0626) with the *P*4*mm* space group [20–21]. The major diffraction peaks at around 22.1°, 31.6°, 38.5°, 44.8°, 45.2°, 55.7°, and 56.0° are indexed as (100), (110), (111), (002), (200), (112), and (211), respectively. These diffractions are used to identify the tetragonal BTO lattice. A distinct peak splitting around 45° corresponding to the Miller indexes (002) and (200) differentiates tetragonal BTO from cubic BTO [20]. Thus, as-prepared BTO nanoparticles are monocrystalline and exhibit a tetragonal lattice structure. Also, there is no sign of any impurity due to the repeated washing of BTO nanoparticles with dilute formic acid solution, which removes common impurities such as BaCO_3_ [22].

XRD pattern of core–shell BTO-PTh nanoparticles shows the characteristic diffractions of tetragonal BTO along with the additional peaks corresponding to certain impurities or by-products formed during the oxidative polymerization of Th. Albeit the crystallinity of core–shell BTO-PTh nanoparticles is slightly affected by the amorphousness of the PTh coating, the tetragonal perovskite structure (indicated by #) is still dominant. The additional diffraction peaks (indicated by *) are attributed to orthorhombic BaSO_4_ [23]. It is a consequence of the leaching of Ba^2+^ ions from BTO nanoparticles during the polymerization reaction [24], which can react with SO_4_^2−^ ions in the solution to form insoluble BaSO_4_. Nevertheless, XRD patterns of BTO and BTO-PTh nanoparticles confirm the dominant tetragonal perovskite structure of the BTO lattice.

**Figure 4:**
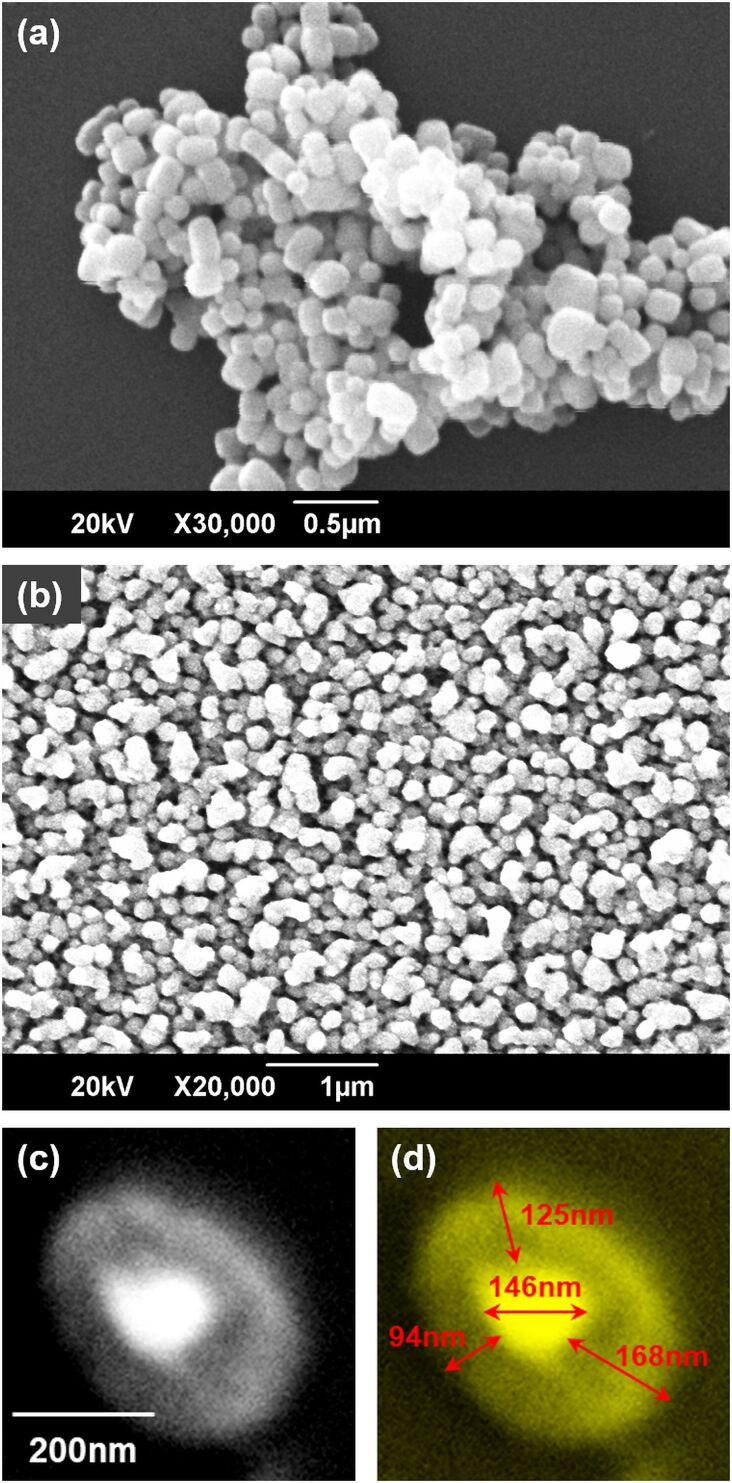
SEM images of the as-prepared BTO nanoparticles (a) and the core–shell BTO-PTh nanoparticles (b). The core–shell structure of BTO-PTh nanoparticles is demonstrated in panels (c, d).

Figure 4a shows a SEM image of as-prepared BTO nanoparticles. Clusters or agglomerates of nanoparticles can be seen with easily identifiable individual BTO nanoparticles. The size of BTO nanoparticles is in the range of 70–150 nm. In contrast, the core–shell BTO-PTh nanoparticles exhibit uniform surface morphology, as shown in Figure 4b. The size of BTO-PTh nanoparticles is in the range of 300–500 nm. The core–shell structure of oval-shaped BTO-PTh nanoparticles is demonstrated in Figure 4c,d. A thick shell of PTh (thickness: 90–170 nm) is formed around BTO nanoparticles, which may comprise more than one layer of PTh. It is assumed that PTh multilayers are held together through π–π stacking interactions between the polymeric chains, which prevent PTh from irreversible slithering out-of-place [25]. Therefore, core–shell BTO-PTh nanoparticles possess a distinct morphology.

**Figure 5:**
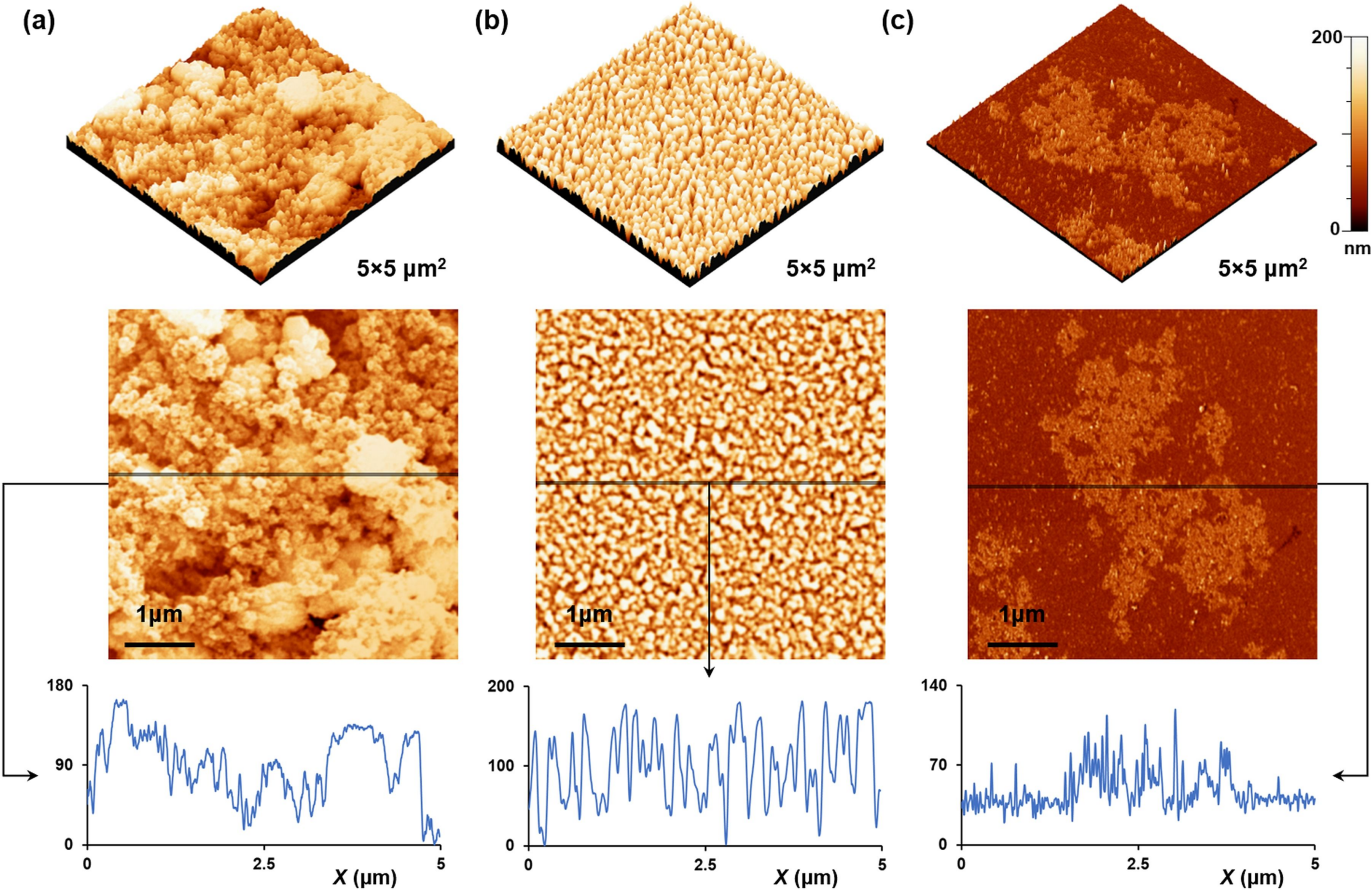
AFM images of the as-prepared BTO nanoparticles (a), BTO-PTh nanoparticles (b), and pristine PTh (c). 3D images showing the surface topography, and 2D images along with surface profiles showing the surface morphology of all samples.

The surface morphology of all samples is further studied using atomic force microscopy, after depositing the samples on quartz wafers. Figure 5 shows the 2D- and 3D-AFM images of BTO, BTO-PTh, and PTh samples along with their surface profiles. The micrographs of BTO nanoparticles show the presence of clusters on the surface. This is in agreement with the SEM image shown in Figure 4a. PTh, on the other hand, exhibits an inhomogeneous surface morphology with large flakes of polymer randomly distributed on the surface. In case of core–shell BTO-PTh nanoparticles (Figure 5b), the surface topography is very consistent with uniformly distributed sub-micrometer particles or agglomerate on the surface. The same is observed in the surface profile of core–shell BTO-PTh nanoparticles, which is monotonous on the height scale. Pristine PTh shows a variable surface profile because of the inconsistent presence of large polymer flakes. BTO nanoparticles also exhibit irregular surface profile, which confirms the occurrence of sub-micrometer clusters and nanoscale particles on the surface.

**Figure 6:**
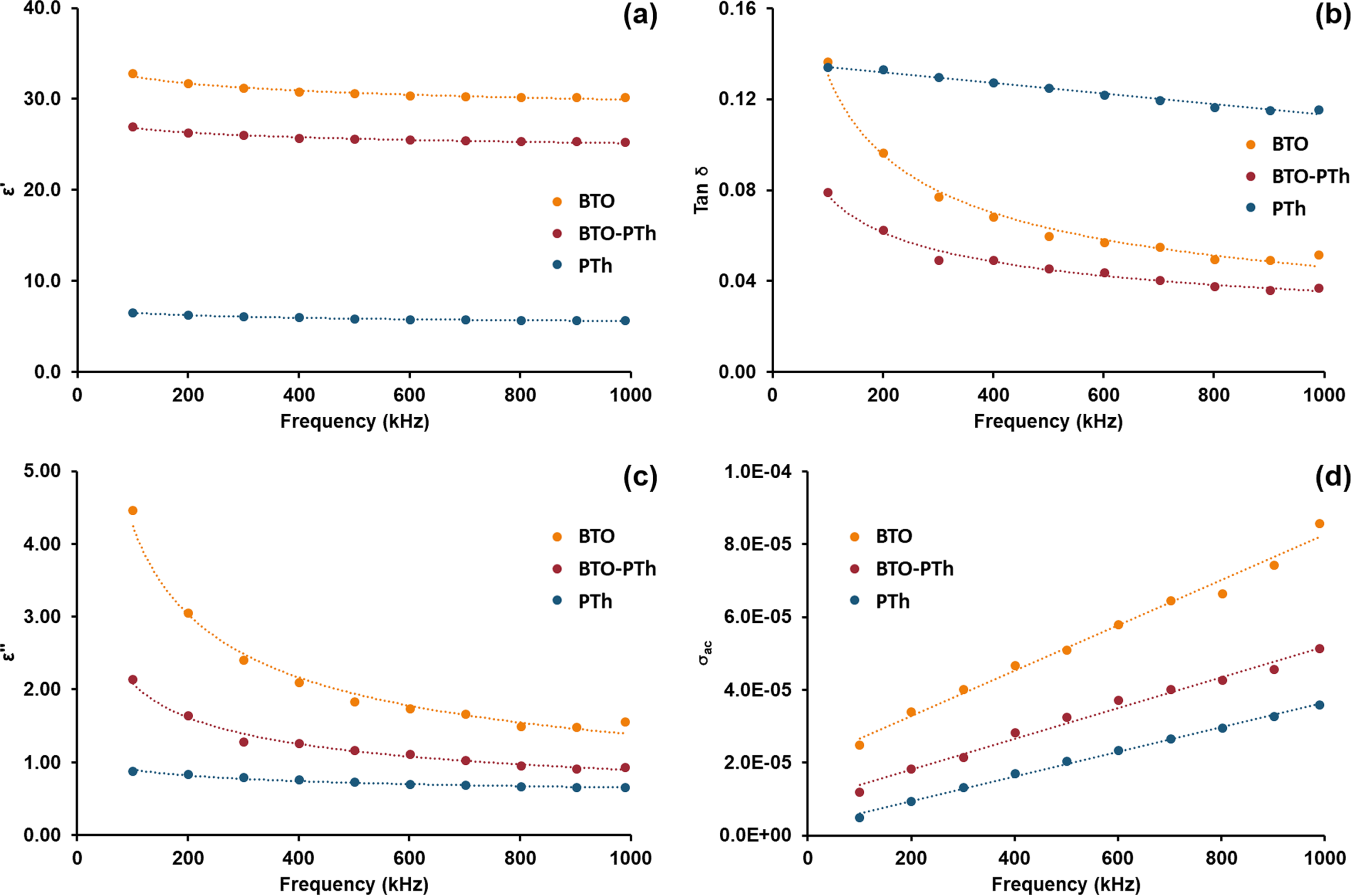
Dielectric properties of the as-prepared BTO nanoparticles, pristine PTh, and BTO-PTh nanoparticles. Permittivity or dielectric constant (a), loss tangent (b), dielectric loss (c), and ac conductivity (d) are plotted as a function of the frequency.

The permittivity or dielectric constant (ε′), loss tangent (tan δ), dielectric loss (ε″), and ac conductivity (σ_ac_) of the synthesized materials are measured as a function of the ac frequency at room temperature. The variation of the frequency-dependent complex dielectric permittivity is shown in Figure 6a. The real part of the complex dielectric permittivity of all three samples exhibits a relatively low frequency dependence in the 100–1000 kHz range. The rate of decreasing permittivity as a function of frequency is in the order of PTh (13.8%) > BTO (7.9%) > BTO-PTh (6.3%). A lower rate of decreasing permittivity can be ascribed to good interfacial compatibility in core–shell BTO-PTh nanoparticles [26]. The permittivity values of BTO, BTO-PTh, and PTh nanoparticles at maximum frequency are 30.2, 25.2, and 5.6, respectively.

The frequency dependence of the dielectric loss tangent is shown in Figure 6b. The dielectric loss tangent represents the energy loss within the dielectric medium. Contrary to the dielectric permittivity, the loss tangent exhibits considerable frequency dependence. The dielectric loss tangent is observed to decrease rapidly in the low-frequency region, while the rate of decrease slows down as the frequency rises. Such a behavior of dielectric materials is widely accepted. In the low-frequency region that corresponds to the high resistivity of grain boundaries, more energy is required for electron hopping, thus, increasing the loss [27–28]. In the high frequency region that corresponds to the higher conductivity, energy required for the hopping of electrons is less and therefore, the loss decreases [27–28].

Dielectric loss is an important part of the total core loss in a dielectric material. Figure 6c shows the dielectric loss of all samples at different frequencies. The dielectric loss of BTO and BTO-PTh nanoparticles also exhibits a substantially high frequency dependence, i.e., ε″ decreases by 50–65% as the frequency increases to 1 MHz. The dielectric loss of BTO nanoparticles is reduced to half by PTh coating in BTO-PTh nanoparticles, which is attributed to conduction loss and smaller interfacial polarization due to good compatibility between the two phases [26]. These results agree well with the calculated ac conductivity of BTO and core–shell BTO-PTh nanoparticles. The ac conductivity as a function of the frequency is shown in Figure 6d. It is observed that BTO-PTh nanoparticles have a lower ac conductivity than BTO, and the ac conductivity increases linearly with the frequency of the applied field. At lower frequencies, the greater resistive influence of grain boundaries results in lower ac conductivity [28–29]. It is important to note that pristine PTh inherently exhibits the lowest ac conductivity and the highest loss tangent. Therefore, core–shell BTO-PTh nanoparticles offer an excellent combination of electrical properties with high permittivity (ε′ = 25.2), very low loss tangent (tan δ = 0.04) and dielectric loss (ε″ = 0.93), and 41% reduced ac conductivity compared to the as-prepared BTO nanoparticles.

**Figure 7:**
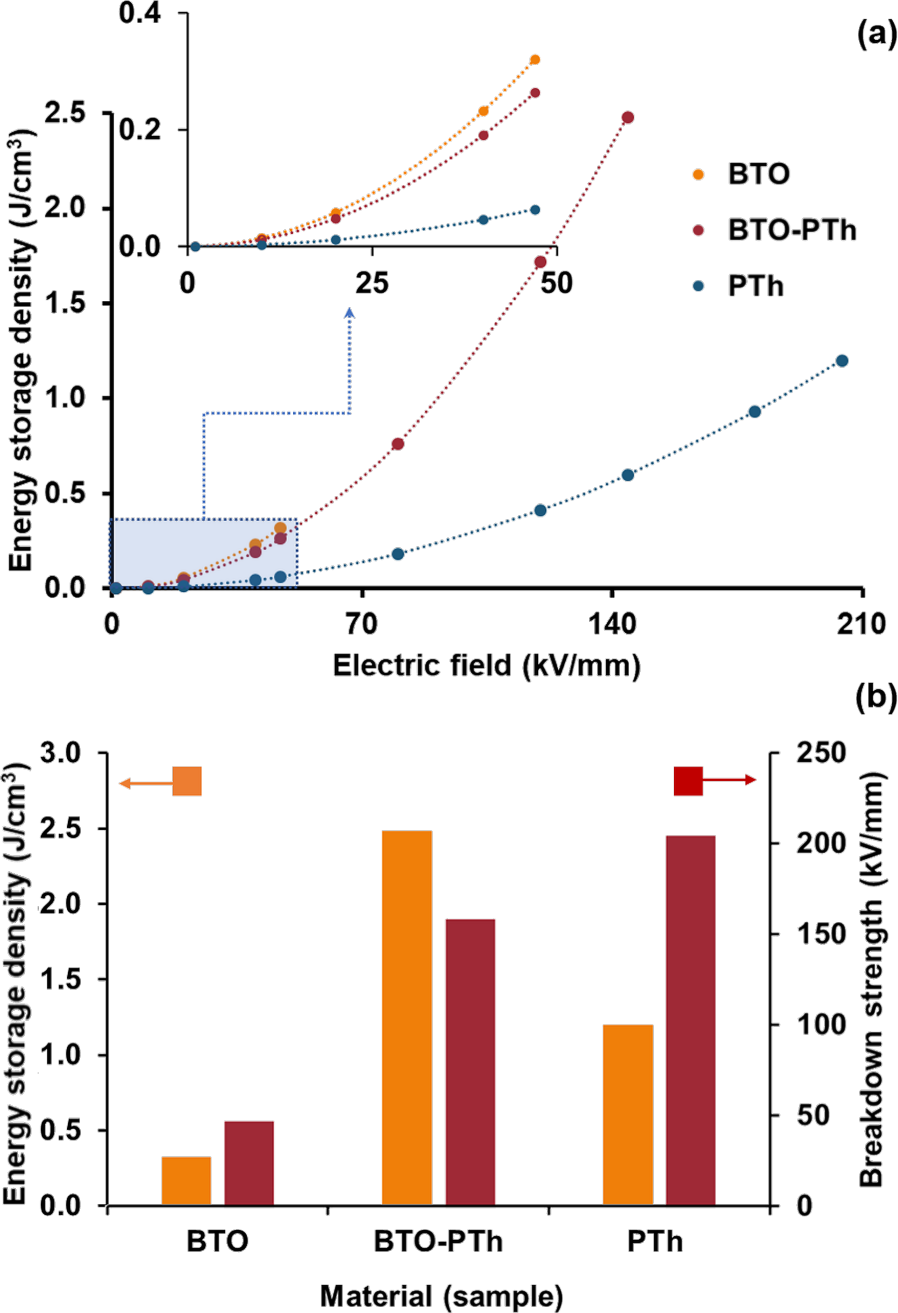
A plot of energy storage density as a function of the electric field strength (a), and the calculated maximum energy storage density and electrical breakdown strength (b) of the different dielectric materials.

Figure 7 shows the breakdown strength (*E*_b_) measured at the room temperature and the calculated energy storage density (*J*) of all samples. The energy density is calculated using Equation 1 [30–31]:

[1] $$J=\frac{1}{2}\varepsilon_{0}\varepsilon^{\prime }E_{\text{b}}{}^{2}.$$

Pristine BTO and PTh nanoparticles exhibit a breakdown strength of 47.0 ± 2.0 and 204.3 ± 15.2 kV/mm, respectively. The PTh coating on BTO nanoparticles results in a 3-fold increase in the breakdown strength (i.e., 144.2 ± 4.9 kV/mm) of BTO-PTh nanoparticles compared to pristine BTO. In turn, the energy storage density of BTO-PTh nanoparticles is calculated as 2.48 J/cm^3^, which is extremely high compared to pristine BTO (0.32 J/cm^3^) and PTh (1.20 J/cm^3^) nanoparticles. It has been investigated that the breakdown strength of BTO-polymer composites is considerably reduced after increasing the BTO content to 30–40 wt % because of the free-charge accumulation at the interface of BTO and polymer [10]. We believe that core–shell structure of BTO-PTh nanoparticles and good interfacial compatibility between the two phases prevent the free-charge accumulation at the interface and, therefore, improve the breakdown strength.

Furthermore, in situ oxidative polymerization of PTh on BTO surfaces allows for the inclusion of 90 wt % BTO, which results in a high dielectric constant. This means that the tremendous increase in the energy storage density of core–shell BTO-PTh nanoparticles is due to the combined effect of high dielectric constant (ε′) and improved breakdown strength (*E*_b_). These results are noteworthy considering the previous examples of BTO-polymer composite dielectric materials. Table 1 offers a comparison of dielectric properties, breakdown strength, and energy storage density between other BTO-polymer composite-based dielectric materials and those described in this work. It shows that core–shell BTO-PTh nanoparticles can be used alone or as reinforcement in various polymer matrices for prospective microelectronic and energy storage applications.

**Table 1:** A comparison of the electrical properties of core–shell BTO-PTh nanoparticles with other BTO-polymer composite dielectric materials reported in literature.

material	dielectric properties			breakdown strength (kV/mm)	energy storage density (J/cm^3^)	ref.

	frequency (Hz)	permittivity (ε′)	loss tangent (tan δ)			

core–shell BaTiO_3_-polythiophene nanoparticles [BTO/PTh = 9:1 w/w]	10^6^	25.23	0.04	144	2.48	this work
core–shell BaTiO_3_@polyaniline/polyarylene ether nitrile nanocomposites [40 wt % BTO@PANI]	10^3^	14.0	0.025	169.8	1.8	[10]
core–shell BaTiO_3_@SiO_2_/polyamic acid [5 vol % BTO@SiO_2_]	10^6^	4.1	0.009	345	2.3	[32]
core–shell BaTiO_3_@Al_2_O_3_/polyvinylidene fluoride [10 vol % BTO@Al_2_O_3_]	10^3^	16.27	0.02	250	4.32	[33]
core–shell polylactic acid@polydopamine@BaTiO_3_ [20 vol % BTO]	10^6^	8	0.01	95	1.52	[34]
core–shell Ag@polydopamine@BaTiO_3_/poly(vinylidene fluoride-*co*-hexafluoropropylene) composites [20 wt % BTO; 2 wt % Ag]	10^6^	≈18	0.16	248	3.15	[35]
core–shell BaTiO_3_@polyamide@poly(methyl methacrylate) [56.7 wt % BTO]	10^6^	39.4	0.028	—	0.03	[36]
core–shell polystyrene/BaTiO_3_ nanocomposites [47.7 vol % BTO]	10^6^	24.27	0.013	1.4	0.027	[37]
core–shell BaTiO_3_-poly(styrene-*co*-vinylbenzyl chloride)/polystyrene composite [75 wt % BTO@polymer]	10^4^	22.3	0.079	95	0.9	[38]
core–shell BaTiO_3_-polystyrene-block-poly(styrene-*co*-vinylbenzyl chloride)/polystyrene composite [75 wt % BTO@polymer]	10^4^	44.7	0.060	222	9.7	

## Conclusion

A simple Cu(II)-catalyzed chemical oxidative polymerization reaction in the presence of BaTiO_3_ nanoparticles is reported for the synthesis of polythiophene-encapsulated BaTiO_3_ nanoparticles as a novel dielectric material. The procedure allows the formation of BTO-rich core–shell-type BTO-PTh hybrid nanoparticles with a BTO/PTh mass ratio of 9:1. We achieved excellent dielectric properties with high permittivity, low dielectric loss, and excellent energy storage density. By PTh encapsulation, it was possible to simultaneously have a high dielectric constant and excellent breakdown strength of BTO nanoparticles, thereby increasing the energy storage density. This type of core–shell BTO-PTh nanoparticles may show great potential for future research and applications regarding energy storage devices.
